# Inhibition of the colony-stimulating-factor-1 receptor affects the resistance of lung cancer cells to cisplatin

**DOI:** 10.18632/oncotarget.10895

**Published:** 2016-07-28

**Authors:** Harvey I. Pass, Carmencita Lavilla, Claudia Canino, Chandra Goparaju, Jordan Preiss, Samrah Noreen, Giovanni Blandino, Mario Cioce

**Affiliations:** ^1^ Division of Thoracic Surgery, Department of Cardiothoracic Surgery, Langone Medical Center, New York University, New York, USA; ^2^ New York University Langone Medical Center, New York University, New York, USA; ^3^ Translational Oncogenomics Unit, Italian National Cancer Institute 'Regina Elena', Rome, Italy; ^4^ Department of Oncology, Juravinski Cancer Center-McMaster University, Hamilton, Ontario, Canada; ^5^ University Campus Biomedico, Rome, Italy

**Keywords:** lung cancer, chemoresistance, CSF-1, CSF-1R, ALDH

## Abstract

In the present work we show that multiple lung cancer cell lines contain cisplatin resistant cell subpopulations expressing the Colony-Stimulating-Factor-Receptor-1 (CSF-1R) and surviving chemotherapy-induced stress. By exploiting siRNA-mediated knock down *in vitro* and the use of an investigational CSF-1R TKI (JNJ-40346527) *in vitro* and *in vivo*, we show that expression and function of the receptor are required for the clonogenicity and chemoresistance of the cell lines. Thus, inhibition of the kinase activity of the receptor reduced the levels of EMT-associated genes, stem cell markers and chemoresistance genes. Additionally, the number of high aldehyde dehydrogenase (ALDH) expressing cells was reduced, consequent to the lack of cisplatin-induced increase of ALDH isoforms. This affected the collective chemoresistance of the treated cultures. Treatment of tumor bearing mice with JNJ-40346527, at pharmacologically relevant doses, produced strong chemo-sensitizing effects *in vivo*. These anticancer effects correlated with a reduced number of CSF-1R^pos^ cells, in tumors excised from the treated mice. Depletion of the CD45^pos^ cells within the treated tumors did not, apparently, play a major role in mediating the therapeutic response to the TKI. Thus, lung cancer cells express a functional CSF-1 and CSF-1R duo which mediates pro-tumorigenic effects *in vivo* and *in vitro* and can be targeted in a therapeutically relevant way. These observations complement the already known role for the CSF-1R at mediating the pro-tumorigenic properties of tumor-infiltrating immune components.

## INTRODUCTION

Lung tumors account for the highest rate of cancer incidence and cancer-related mortality worldwide [1, 2]. Chemoresistance is a major obstacle to the therapeutic management of lung cancer [3, 4]. Resistance to therapy can be ascribed to multiple factors, both intrinsical to tumor cells (refractory cell cycle status, metabolic switches, expression of drug effluxing factors, unique redox potential) [5–7] and derived from the tumor microenvironment (infiltrating stromal cells) [8–11]. In such a context, it is also emerging that, within solid tumors, there exist functionally distinct cell subpopulations, whose size and distribution are dynamically modulated during the history of the disease and strongly influenced by therapy-induced stress [12]. Single cell RNAseq approaches have recently provided further support to this view [13–16]. This tumor heterogeneity can drive the progression of the disease and can impinge on chemoresistance [17–25]. Survival signals can be provided by profound changes in the secretome of the treated tumor cells, with the release of growth factors, cytokines and other mediators, involved in the downstream signaling leading to chemoresistance [11, 26–30]. In line with this, receptor tyrosine kinases (RTK), among the most frequently modulated molecular hubs within chemoresistant tumors [31], may appear as ideal candidates to transduce stress-induced micro-environmental changes into an altered distribution of tumor cell subpopulations [32]. Thus, identification of ligands released by tumor cells acquiring chemoresistance and/or inhibition of the downstream RTKs is an appealing theatre of investigation [26]. The Colony-Stimulating-Factor Receptor-1 (CSF-1R) is a type III receptor tyrosine kinase (RTK) primarily known as responsible for promoting the differentiation, survival and homing of the monocyte–macrophage cell lineages [33]. CSF-1R may function as an oncogene [34] in a src- dependent and independent way [35, 36]. It was also shown to be capable of direct oncogenic transcriptional activity [37] and to sensitize breast cancer cells to therapy [38]. *In vivo*, elevated expression of CSF-1R has been described in tumors of epithelial origin and shown to correlate in some cases with adverse prognosis [39–44]. In breast cancer and renal cell carcinoma (RCC) autocrine signaling by CSF-1R was demonstrated *in vivo* and was modulated by TGFb1 and EGF, respectively [43, 45]. Finally, lineage inappropriate expression of CSF-1R into Hodgkin's lymphoma cells [46] and mesothelioma cells [47] is oncogenic. Here, we started from the observation that the secretome of cisplatin treated lung cancer cells is enriched for the CSF-1R ligand, CSF-1, which was secreted by almost all the lung cancer cell lines in our collection. This correlated with the persistence and survival of the CSF-1R expressing cell subpopulations to cisplatin treatment, which relied on the presence of both the receptor and its ligand, as shown by siRNA approaches. We tested whether this observation could be exploited therapeutically by means of a “clinical trial grade”, investigational CSF-1R TKI inhibitor [48, 49]. In detail, treatment with the CSF-1R TKI affected the clonogenicity and the 3D growth of the lung cancer cells. Despite the CSF-1R^pos^ cells represented a minor fraction of the cells within the culture, knocking down the receptor or inhibiting its kinase activity, at pharmacologically relevant doses, affected the chemoresistance of the whole unfractionated culture *in vitro*. This correlated with downregulation of several stem cell markers, chemoresistance genes and EMT markers. We observed similar effect when using an unrelated CSF-1R TKI inhibitor, BLZ-945. Similarly to what observed *in vitro*, treatment of mouse xenografts (NCI-H1299) with the TKI affected tumor growth and sensitized the tumors to clinically relevant doses of cisplatin. This latter effect correlated with changes in the number of the CSF-1R expressing cells represented in the excised tumor masses treated with the inhibitor, in combination with cisplatin.

## RESULTS

### Multiple lung cancer cell lines contain CSF-1R expressing cells

**Figure 1:**
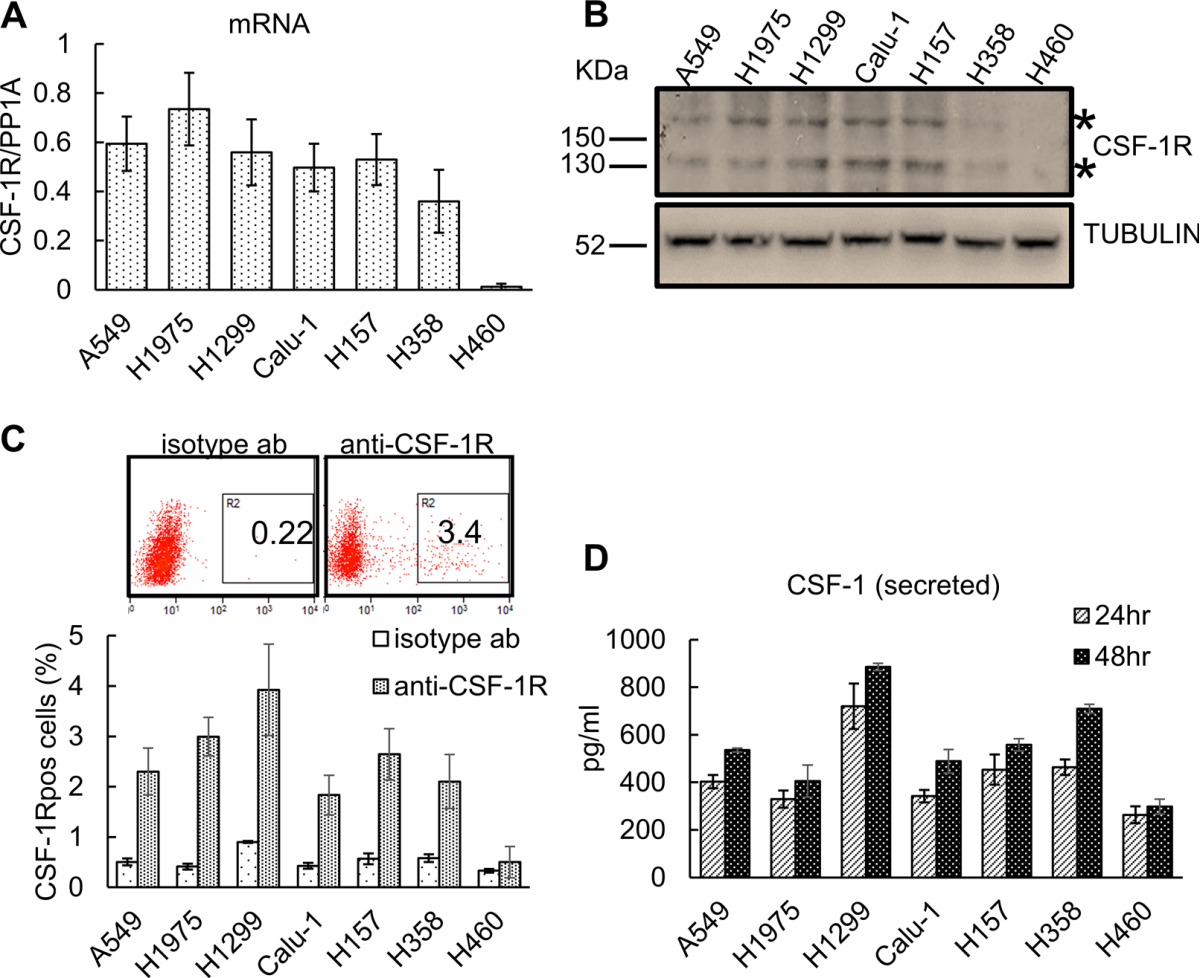
Lung cancer cell lines express CSF-1R and CSF-1 and contain CSF-1R^pos^ cell subpopulations (**A**) Quantitative PCR. Histograms reporting the levels of CSF-1R mRNA (normalized to the PP1A mRNA) as assessed in 7 representative lung cancer cell lines. Mean ± SE of three independent experiments. (**B**) Western blotting with anti-CSF-1R antibodies of cell lysates from the indicated lung cancer cell lines. *asterisks indicate the two main species of the CSF-1R (165 kDa and 135 kDa, respectively). (**C**) Upper panel. Representative FACS dot plots of H1299 cells stained with a anti-CSF-1R antibody (right) or with an isotype matched antibody (as a background control, left). The percentage of the gated cells is shown. Lower panel. Histograms reporting the percentage of CSF-1R^pos^ cells as assessed by FACS. Mean ± SE of three independent experiments. (**D**) The lung cancer cell lines secrete CSF-1. ELISA assay. Levels of CSF-1 in the media conditioned from the indicated lung cancer cell lines and harvested at 24 hr and 48 hr, respectively. The levels of CSF-1 in the cell free medium were subtracted as a background control. Mean ± SE of two independent experiments.

We tested seven representative lung cancer cell lines (Table 1) for the expression of CSF-1R and its ligands, CSF-1 and IL-34 (Figure 1). Quantitative PCR revealed that all but one of the representative cell lines expressed detectable amounts of the receptor mRNA (Figure 1A). Western blotting with anti-CSF-1R antibodies confirmed that a low but detectable amount of the extracellular (Mr 165,000) and intracellular (Mr 135,000) forms of the receptor in six out of seven cell lines (Figure 1B). FACS analysis after staining with anti-CSF-1R antibodies revealed the existence of distinct cell subpopulations expressing the receptor (range 2–5%, Figure 1C), compatible with the existence of a fraction of the cells expressing CSF-1R on the cell membrane, at steady state. In agreement with previously published work [50], we did not observe expression of the receptor in the NCI-H460 cells (Figure 1A–1C). We also assessed the expression of the CSF-1R ligands (CSF-1 and IL-34) by quantitative PCR and ELISA, respectively, and this revealed that most of the cell lines expressed CSF-1 mRNA, with three of them (A549, H1299 and Calu-1) expressing barely detectable amounts of IL-34 mRNA (Supplemantary Figure S1A). ELISA assay of conditioned medium (72 hrs) from the analyzed cell lines revealed detectable levels of secreted CSF-1 and no detectable IL-34 (Figure 1D and data not shown, respectively). Since almost all of the lung cancer cell lines in our collection exhibited CSF-1/CSF-1R expression, we focused on the CSF-1/CSF-1R duo for the subsequent experiments. Thus, multiple lung cancer cell lines expressed detectable amount of CSF-1R and its ligand CSF-1, thus suggesting that they are capable of CSF-1R signaling.

**Table 1:** Main features of the lung cancer cell lines used in this study

	Histotype	origin	Morphology	CSF-1Rpos cells (%)
A549	lung carcinoma	primary site	epithelial	3.4 ± 0.3
NCI-H1299	NSCLC	metastatic	epithelial	3.9 ± 1.1
NCI-H157	NSCLC	metastatic	epithelial	2.9 ± 0.6
CALU-1	Epidermoid Carcinoma	pleural effusion (metastasis)	epithelial	1.8 ± 0.4
NCI-H1975	AdenocarcinomaNSCLC		epithelial	2.8 ± 0.6
NCI-H358	bronchioalveolar carcinoma; non-small cell lung cancer	metastatic site	epithelial	3.8 ± 0.2
NCI-H460	large cell lung cancer	pleural effusion (metastatic)	epithelial	0.8 ± 0.3

### CSF-1R expression may confer resistance to chemotherapy-induced stress

**Figure 2:**
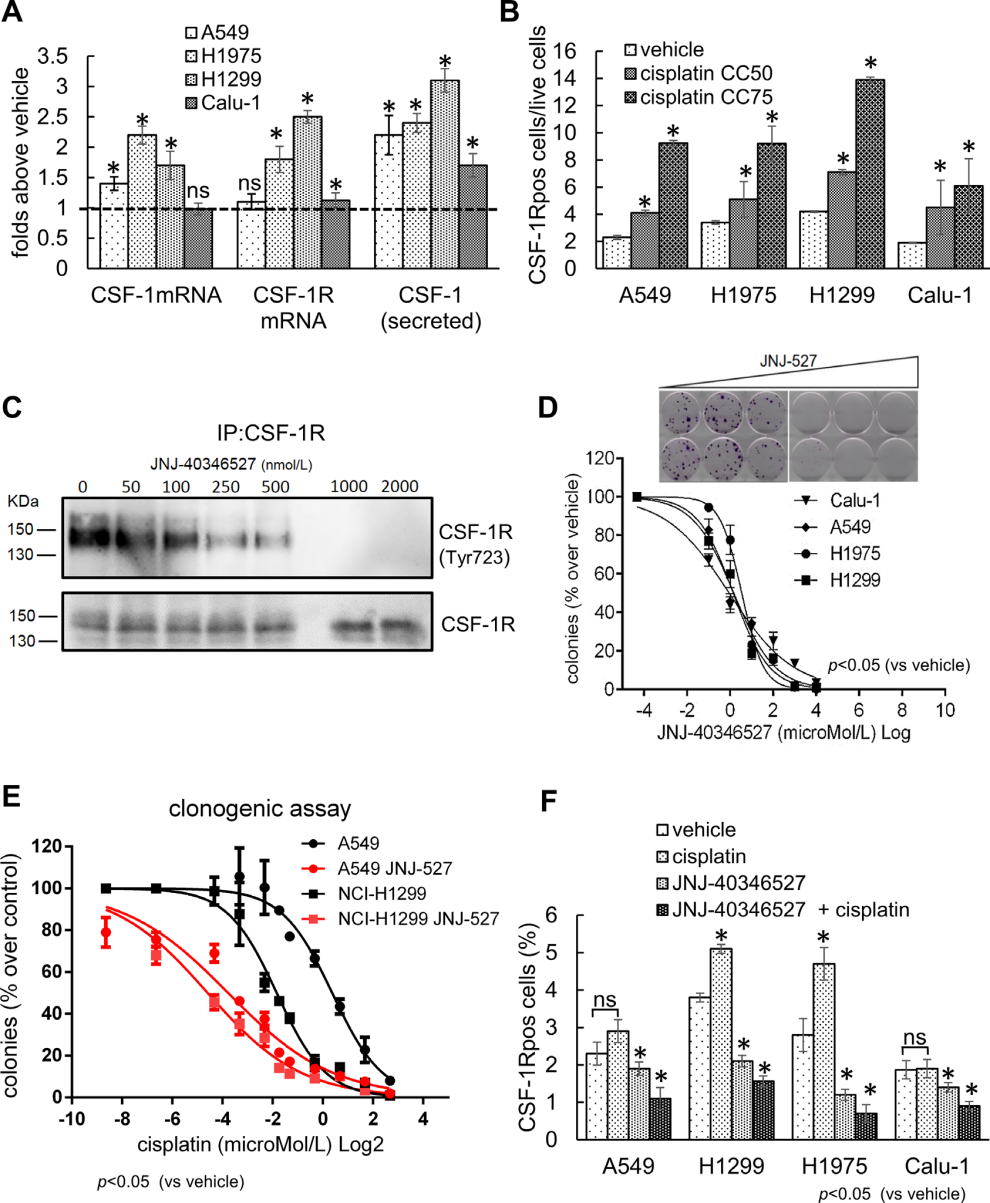
The expression of CSF-1R and its ligand influence the resistance to cisplatin treatment (**A**) Cisplatin treatment increases mRNA and protein levels of CSF-1 and CSF-1R. Histograms reporting the mRNA levels of CSF-1 and CSF-1R, in cells harvested at 24 hr (quantitative PCR), and the protein levels of CSF-1 in medium conditioned for 48 hr (ELISA assay), from the indicated cell lines treated with cisplatin at the CC_50_ doses. (**B**) The CSF-1R expressing cells survive chemotherapy-induced stress. Upper panel. Representative FACS dot plots showing the percentage of CSF-1R^pos^ in H1299 cells treated with cisplatin at the CC_50_ and CC_75_ for 72 hrs (gated). Gated cells and isotype controls are further illustrated in Supplementary Figure S1B. Lower panel: histogram bars reporting the percentage of CSF-1R_pos_ cells in the indicated cell lines. In the lower panel, the mean ± SE of two independent experiments is reported. (**C**–**D**) CSF-1R inhibition affects the clonogenicity and resistance to cisplatin of lung cancer cell lines. (C) Western blotting of CSF-1R immunoprecipitates stained with the indicated antibodies from H1299 cells treated with increasing concentrations of JNJ-40346527. (D) Upper panel. Representative micrographs of the colonies formed by H1299 cells treated with increasing doses of JNJ-40346527. Lower panel. Histograms reporting the mean ± SE of the colonies formed by 4 representative cell lines in three independent experiments. (**E**) CSF-1R inhibition affects the resistance of lung cancer cell lines to cisplatin. Graphs reporting the percentage of colonies formed by A549 and H1299 cells treated with JNJ-40346527 at the CC_25_ doses determined in 2D and with the indicated doses of cisplatin. Mean ± SE of three independent experiments. (**F**) JNJ-40346527 treatment modulates the number of CSF-1R^pos^ cells. Histograms reporting the number of CSF-1R^pos^ cells (as assessed by FACS) in the H1299 cells treated for 96 hrs at the previously determined CC_50_ dosages for both cisplatin and JNJ-40346527. Mean ± SE of three independent experiments is reported. *= *p* < 0.05.

Ectopic expression of CSF-1R was shown to convey pro-tumorigenic properties, to breast cancer and mesothelioma cells [36, 47, 51]. Thus, we evaluated whether the expression of CSF-1R may confer resistance to chemotherapy-induced stress, a frequent driver of lung cancer progression. First, quantitative PCR of mRNA obtained from 4 representative lung cancer cell lines (H1975, NCI-H1299, A549 and Calu-1) revealed that both the receptor and the ligand CSF-1 mRNAs were steadily increased by cisplatin treatment (24 hrs, CC_50_) (Figure 2A). Analysis of the conditioned medium from the same cells revealed increased protein levels of CSF-1 in the medium of the cisplatin treated ones (Figure 2A). Thus, lung cancer cell lines could upregulate CSF-1 mRNA and protein and secrete CSF-1 in the medium after cisplatin treatment. Interestingly, these findings correlated with the persistence of CSF-1R expressing cells in the cisplatin treated samples, as assessed by FACS analysis of the cells treated with CC_50_ and CC_75_ doses of the drug, for 72 hrs (Figure 2B, and Supplemantary Figure S1B–S1C). Thus, CSF-1R expression identified a cell subpopulation capable of surviving cisplatin treatment.

In order to causally link the expression of CSF-1 and CSF-1R with the persistence of the CSF-1R expressing cells after cisplatin treatment, we used siRNAs against either CSF-1R or CSF-1 and we evaluated the effect of depleting the receptor/ligand on the clonogenic capacity of the cells, both at the steady-state and after cisplatin treatment (Supplemantary Figure S2A–S2B). We observed a significantly impaired colony formation in the H1299 and H1975 cells transfected with siRNAs directed towards CSF-1 or CSF-1R (as compared to scrambled control). Such effect was strongly increased by cisplatin treatment at subtoxic doses (CC_25_) (Supplemantary Figure S2B). Lastly, the effect of knocking-down CSF-1R on the clonogenicity of the lung cancer cells was partially rescued by transfecting H1299 and H1975 cells with an expression vector coding for a ligand independent, constitutively active CSF-1R receptor, the *CSF-1R* L301S/*Y969F* [52, 53] (Supplemantary Figure S2B).

To translate the above findings into a more clinically relevant setting, we evaluated the effect of a ”clinical trial” grade CSF-1R tyrosine kinase inhibitor (TKI) (JNJ-40346527) [48, 49] on the clonogenicity of four representative lung cancer cell lines. First, we found that treatment with the TKI revealed a dose-dependent effect of the JNJ-40346527 treatment on the amount of Tyr^723^ phosphorylated CSF-1R (Figure 2C), with a concomitant effect on the number of the formed colonies, at submicromolar doses (Figure 2D, upper and lower panels). Next, we tested whether the TKI treatment would sensitize the cells to the effect of cisplatin. Co-treatment of the cells with increasing doses of cisplatin and JNJ-40346527, the latter at the previously determined CC_25_ doses (Table 2), revealed a strong potentiation of the effect of the cisplatin (Figure 2E).Notably, we observed very similar chemosensitizing effects when using an unrelated CSF-1R TKI, the BLZ-945 [54, 55] (Supplementary Figure S3A). Thus, inhibition of CSF-1R could impair both clonogenicity and chemoresistance of the lung cancer cell lines. This echoed the persistence of the CSF-1R^pos^ cells in the cisplatin-treated samples and showed that inhibiting CSF-1R in a subset of cells affected the collective resistance of the cell line to chemotherapy-induced cell death.

**Table 2:** CC_50_ of the mentioned compounds, as assessed by clonogenic assay

	Cisplatin micromol/L	JNJ-40346527 micromol/l	BLZ-945 (micromol/l)
A549	3.5 ± 0.6	0.41 ± 0.5	
NCI-H1299	0.6 ± 0.12	0.33 ± 0.09	0.48 ± 0.12
NCI-H157	2.1 ± 0.5	0.39 ± 0.1	
CALU-1	3.1 ± 0.8	0.26 ± 0.12	
NCI-H1975	3.5 ± 0.6	0.24 ± 0.07	0.33 ± 0.09
NCI-H358	1.9 ± 0.3	2.5 ± 0.11	
NCI-H460	1.2 ± 0.4	>10	

### CSF-1R inhibition affects the number of chemoresistant CSF-1R^pos^ cells

Since the chemosensitizing effect of the TKI could take place through a change in the number of the CSF-1R^pos^ cells, we evaluated, by FACS, the number of CSF-1R^pos^ cells after 96 hrs treatment at the previously determined CC_50_ doses of JNJ-40346527 and cisplatin, alone or in combination (Table 2) (Figure 2F). As previously reported in Figure 2B) the CSF-1R^pos^ cells survived cisplatin treatment. Treatment with JNJ-40346527 significantly reduced the number of CSF-1Rpos cells (*p* < 0.05); however, this effect was much stronger when both cisplatin and the TKI were co-administered (Figure 2F). A similar effect on the CSF-1R^pos^ cells was observed when either CSF-1 or CSF-1R were depleted by siRNAs (Supplementary Figure S2C), implying that a reduced number of the CSF-1R expressing cells, due to lower levels of the ligand/receptor or to inhibition of its kinase activity may underlie the chemosensitizing effects of the TKI.

### The CSF-1R TKI affects the sphere forming ability of the treated lung cancer cells

**Figure 3:**
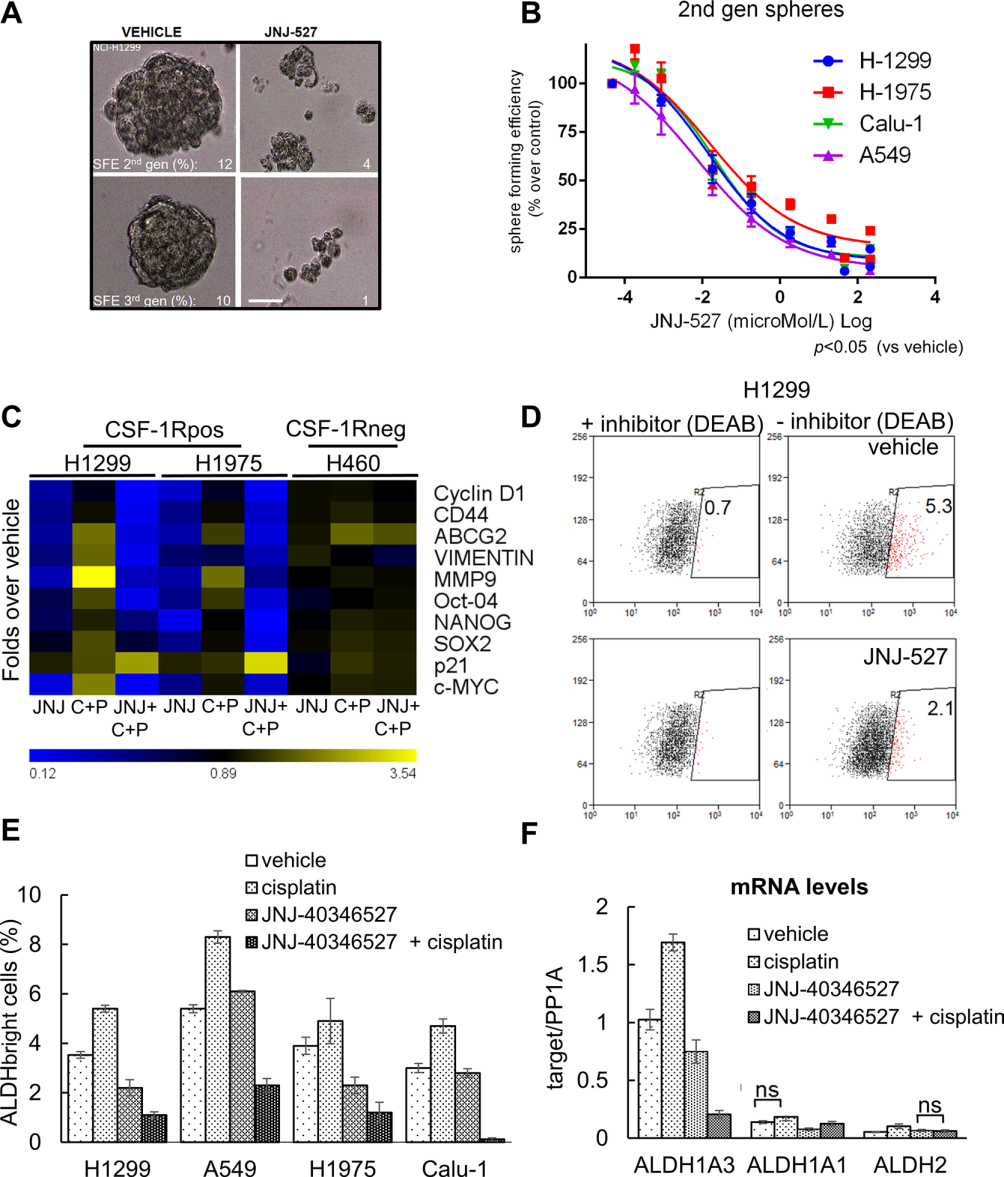
The inhibition of CSF-1R affects some protumorigenic features of lung cancer cells (**A**) CSF-1R inhibition affects the propagation of Sphere Forming Cells in 3D cultures. Representative micrographs of H1299 cultures serially propagated in no serum, no adhesion conditions, in the presence of growth factors. The Sphere Forming Efficiency (SFE) was calculated as the percentage of formed spheres/seeded cells scale bar: 100 micrometers. (**B**) Graph reporting the sphere forming efficiency (SFE) of 4 representative cell lines treated with increasing doses of JNJ-40346527. Mean ± SE of two independent experiments. (**C**) Inhibition of CSF-1R affects the levels of cancer related mRNAs. Heat map. Quantitative PCR. mRNA levels of the indicated genes from H1299 and H1975 cells (expressing CSF-1R) and from H460 cells (not expressing the receptor), treated with vehicle or JNJ-40346527 for 24 hrs , alone or in combination with cisplatin(CC_50_). Please note that at 24 hrs of treatment no significant cell death was observed at the time of harvesting (24 hrs) (data not shown). (**D**–**F**) Inhibition of CSF-1R affects the number of chemoresistant ALDH^bright^ cells. (D) Representative dot plots of H1299 treated for 72 hr with cisplatin in the presence of vehicle or JNJ-40346527 (upper and lower panel, respectively). The high ALDH expressing cells were defined as the cells that displayed greater fluorescence (right panels) compared with a control staining reaction containing the ALDH inhibitor, DEAB (diethylaminobenzaldehyde) (left panels), upon addition of the synthetic ALDH substrate BAAA. (E) Histogram reporting the number of ALDH^bright^ cells from four representative cell lines after the indicated treatments for 96 hrs. Background staining obtained with DEAB-treated cells was subtracted for each sample analyzed. The mean ± SEM of three independent experiments is reported. (F) Quantitative PCR. Levels of mRNA of the indicated ALDH isoforms in H1299 cells treated with JNJ-40346527(CC_50_), in presence or absence of cisplatin, for 24 hrs. PP1A was used as internal control. The mean ± SE of two independent experiments is reported. Statistical differences were indicated when not significant (ns). Very similar results were obtained with H1975 cells (data not shown).

Growth of cells in anchorage independency, at a clonal density and in serum free media enriches for progenitor-like cell subpopulations expressing stem like markers and chemoresistance genes [56]. We thus evaluated the ability of JNJ-40346527 treatment to affect the Sphere Forming Efficiency (SFE) of the treated lung cancer cell lines. More specifically we evaluated the effect of JNJ-40346527(at the previously determined CC_50_) on the formation of second and third generation spheres, obtained by sequential passaging of the originating cell subpopulations in the above mentioned conditions. This revealed a very pronounced effect of the TKI on the Sphere-Forming-Efficiency (SFE) of 4 representative cell lines, which all responded with similar kinetics to the effect of the TKI (Figure 3A–3B). To detail these observations, we evaluated whether the effect of the JNJ-40346527 on the SFE was accompanied by modulation of EMT/stem like markers and chemoresistance genes, as assessed by quantitative PCR. This revealed a significant downregulation of CD44, OCT4, SOX2, NANOG, VIMENTIN, MMP-9 and ABCG2 in the TKI-treated H1299 and H1975 cells, respectively. Additionally, we observed that the JNJ-40346527 treatment increased the levels of p21 and decreased the levels of Cyclin D1 and c-MYC (Figure 3C, heat map) (*p* < 0.05). Notably, the promoters of the latter genes were recently shown to be bound by CSF-1R in SKBR3 breast cancer cells [37]. Increased levels of p21 correlated with the anti-clonogenic effects observed in the JNJ-40346527 treated samples. The combined treatment (TKI + cisplatin) strongly increased the levels of p21 and affected to a much higher extent the levels of the EMT/chemoresistance genes (Figure 3C). We observed very similar effects when using the unrelated BLZ945 compound (Supplementary Figure S3B). All these mentioned changes were globally attenuated in H460 cells after treatment with the JNJ-40346527, alone or in combination with cisplatin (Figure 3C). As shown in Figure 1, the H460 cells were devoid of detectable CSF-1 /CSF-1R (Figure 1). This suggests that, at least in part, the observed gene expression changes may derive from direct inhibition of CSF-1R signaling.

### Treatment with the CSF-1R TKI affects the number of chemoresistant ALDH^bright^ cells

Further, we evaluated the effect of the JNJ-40346527 on the Aldehyde Dehydrogenase (ALDH) activity, expressed in lung cancer cell lines and specimens [57, 58]. The ALDH is a detoxifying enzyme whose expression provides stress resistance and identifies therapy-resistant cell subpopulations in multiple tumors [59–62]. FACS analysis revealed that four out of four of the representative lung cancer cell lines contained ALDH^bright^ cells and exhibited increased number of ALDH^bright^ cells upon cisplatin treatment, according to the chemoresistant nature of the ALDH expressing cells (96 hrs, *p* < 0.05; Figure 3D). Treatment with the JNJ-40346527 (at the CC_50_) affected significantly the number of ALDHbright cells and this effect was much stronger when the TKI was administered to the cells in combination with cisplatin (Figure 3E). Lack of inducible ALDH activity may partially explain the chemosensitizing effect of the JNJ-40346527 treatment. Indeed, quantitative PCR analysis revealed that the mRNAs of two major ALDH isoforms expressed in the lung cancer cell lines, namely ALDH1A3 and ALDH1A1 [57, 58, 63], were significantly reduced by the TKI treatment, which strongly discouraged their induction by cisplatin (*p* < 0.05) (Figure 3F). The mRNA levels of the ALDH2 isoform, not known to confer resistance to therapy, were unaffected by both cisplatin and TKI treatment. Thus, the CSF-1R inhibition may strongly impinge on survival and resistance of progenitor-like population in the targeted lung cancer cell cultures and this further supports the observed chemosensitizing effects.

### CSF-1R inhibition affects tumor growth *in vivo*

**Figure 4:**
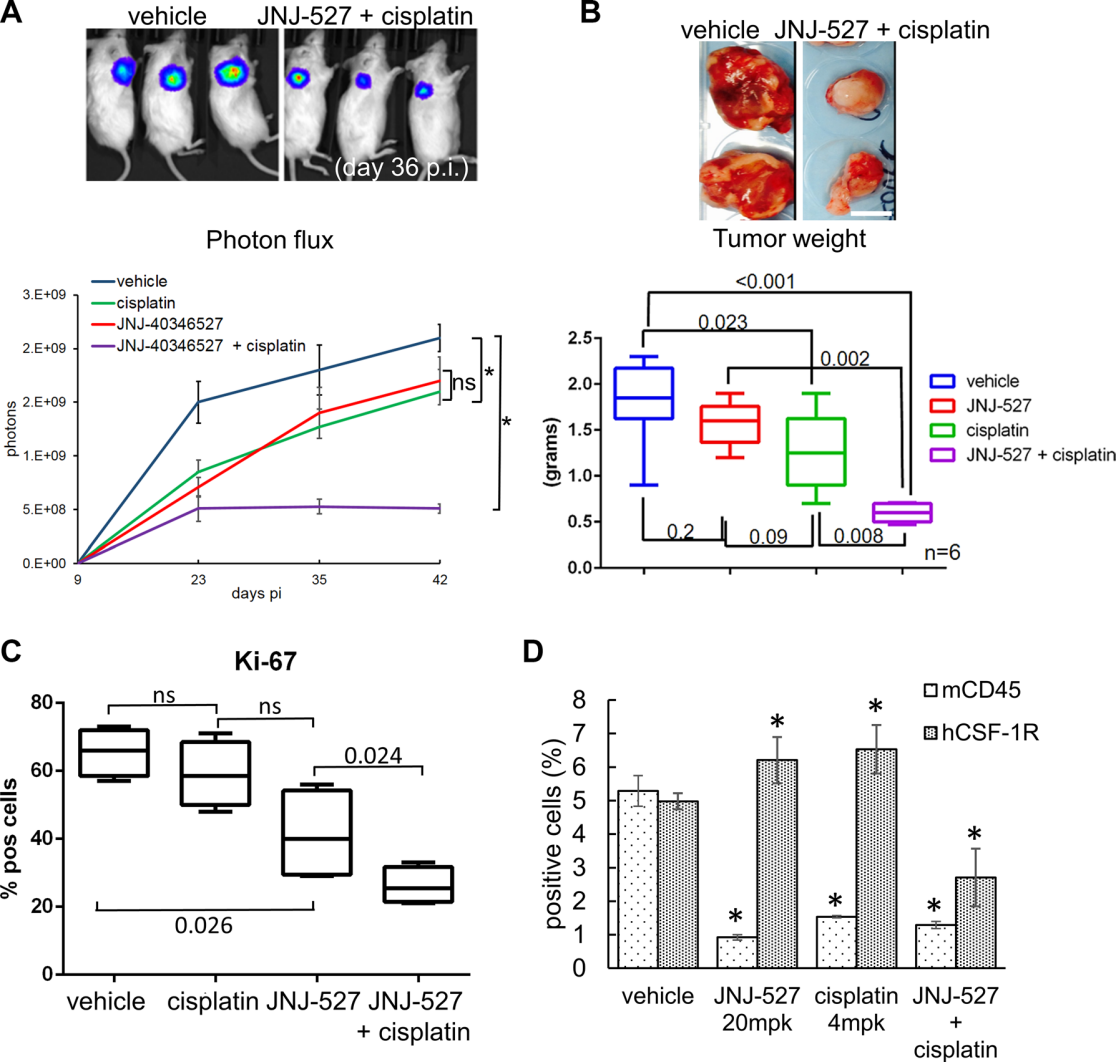
CSF-1R inhibition affects tumor growth (**A**) Upper panel. Representative micrographs. D-Luciferin (150 mg/kg) was injected intraperitoneally and anesthetized mice were imaged at day 36 after cell injection (p.i). Lower panel. Graph reporting the averaged photons emitted in time (day 9, 23, 35, 42 p.i.) for each group. Average ± SE reported for each group.*p < 0.05. (**B**) Upper panel Representative micrographs of the tumors excised at day 42 p.i. from vehicle-(left panel) and cisplatin+ JNJ-40346527 (right panel) -treated mice. Scale bar: 10 millimeters. Lower panel. Graph reporting the dry tumor weight of the excised tumors at day 38 p.i from all of the groups. Mean ± SE for each group of mice is reported. *p-value*s are reported for each paired group. (**C**) The CSF-1R TKI affects the proliferation of the treated tumors. Left panel. Single cells obtained from the disaggregated and pooled tumors were cytospun and stained with an anti-Ki67 antibody. Histograms showing the mean ± SE of Ki-67^pos^ cells for each group of treatment. (**D**) Target engagement in the JNJ-40346527 treated tumors. The CSF-1R TKI targets both human CSF-1R^pos^ cell and murine CD45^pos^ cells. Histogram reporting the percentage of human CSF-1R^pos^ cells and the percentage of murine CD45^pos^ cells as assessed by FACS staining of the disaggregated tumors. Mean ± SE for each group of mice is reported.

The Sphere Forming Assay is considered by some as a surrogate of tumor initiation *in vivo* [64] and thus our previous observations may unravel a potential usefulness of the CSF-1R TKI *in vivo*. This encouraged us to test the JNJ-40346527 in an *in vivo* setting. To this aim, we established a tumor xenograft in NOD-SCID mice injected with luciferase expressing- H1299 cells (Figure 4). Treatment started when tumors reached ≥ 100 mm^3^ in volume (day 14). Mice were administered JNJ-40346527 (20 mg/Kg, by oral gavage, QD, for 16 days, starting at day 14), alone or in combination with cisplatin (4 mg/Kg, intraperitoneal at day 16 and 23) (Supplemantary Figure S4A). Intravital imaging revealed that the JNJ-40346527 treatment affected the growth of the implanted tumors with an effect comparable to that of cisplatin alone (Figure 4A upper and lower panels: *p* < 0.05 vs vehicle). However, when the TKI was combined with chemotherapy, the effect was much stronger than the single treatments (Figure 4A *p* < 0.05), suggesting a synergistic effect and reminiscing the *in vitro* observations. Evaluation of the size and dry weight of the excised tumors at day 48 pi confirmed the imaging data and revealed a strongly decreased weight of the tumors excised from the mice co-administered with JNJ-40346527 and cisplatin (Figure 4B, upper and lower panels, respectively). Further, staining of cytospin sections from the disaggregated and pooled tumors (*n* = 4), with the proliferation marker Ki-67, showed a markedly reduced number of Ki-67 expressing cells, after the combined treatment, with the JNJ-40346527 and cisplatin exhibiting weak activity when administered as single agents to the tumor bearing mice (Figure 4C). In general, both the cisplatin and JNJ-40346527 treatment affected the body weight of the mice. This was a reversible phenomenon since the mice recovered their weight after the treatments were stopped (Supplemantary Figure S4B).

### The effect of the JNJ-40346527 on tumor growth correlated with target engagement

Consistent with the changes in the CSF-1R^pos^ cells observed upon *in vitro* treatment with JNJ-40346527 and cisplatin, we evaluated the number of CSF-1R^pos^ cells in the tumors disaggregated and pooled from the treated mice (*n* = 4) at the end of the study (Figure 4D). Staining with anti-CSF-1Rantibodies revealed a strong reduction of the number of CSF-1R^pos^ cells within the tumors of mice administered with both JNJ-40346527 and cisplatin (as compared to vehicle-treated tumors)(*p* < 0.05) (Figure 4D). No change or a slight increase of the CSF-1Rsignal was observed when the single treatments were administered (Figure 4D). Such an effect may be explained by the reported upregulation of the receptor when targeted by TKI [65] (for the TKI treatment) and with the resistance of the CSF-1R_pos_ cells to cisplatin, as observed *in vitro*. The effect of the CSF-1RTKI on tumor growth did not correlate with changes in the number of host-derived CD45^pos^ cells. The cell line employed for the xenograft studies are virtually devoid of immune-competent cells and NOD-SCID mice represent a deeply immune-compromised microenvironment. Despite this, we could not exclude that the inhibition of tumor growth may result from a TKI-mediated effect on residual immune components of the microenvironment. Thus, we evaluated the percentage of murine CD45^pos^ cells within the treated tumors by FACS (*n* = 4 tumors) (Figure 4D). This showed that the number of CD45_pos_ cells was strongly reduced in the JNJ-40346527 treated tumors (Figure 4D, left panel). However, only when the number of murine CD45_pos_ and human CSF-1R_pos_ cells were concomitantly reduced we observed anticancer effects, suggesting that depletion of the host CD45_pos_ cells was not the main factor underlying the anticancer effects of CSF-1R inhibition, in this experimental system.

## DISCUSSION

The data shown in this work are in line with an emerging trend in cancer studies: that, within a tumor mass, specialized cell subpopulations acquire distinct functional traits, in a very dynamic, stress dependent way. This is accompanied by the emergence of molecular features ultimately impinging on tumor progression, such as chemoresistance. In line with the observation that RTK are among the most dynamic targets contributing to tumor heterogeneity, as shown by single cell RNA-seq studies [66], here we provide evidence for the existence of cisplatin-resistant, lung cancer cell subpopulations expressing the Colony Stimulating Factor Receptor −1 (CSF-1R). We show that the CSF-1R inhibitor JNJ-40346527 (and the unrelated BLZ945) exerted anticancer effects by affecting the number and function of the CSF-1R^pos^ cells after cisplatin treatment. Thus, it counteracted clonogenicity, induction of aldehyde dehydrogenase mRNAs upon cisplatin treatment and decreased the expression of EMT/stem-like markers and that of ABCG2. All this supports the chemosensitizing effects observed *in vivo*.

The potential of CSF-1R to drive tumorigenic properties has been already evoked in several reports in the past, including lung cancer [67]. CSF-1R was shown, in more mechanistic studies, to trigger discohesive features to immortalized breast cells and resistance to pemetrexed to mesothelioma cells [35, 36]. This echoes the original studies by Roussel and colleagues which first demonstrated that the CSF-1R could transform fibroblasts *in vitro* [68, 69]. Here, we believe to provide, in an unprecedented manner, that lung cancer cell-specific expression of the receptor may be functionally relevant and can be therapeutically exploited. Thus, this work, together with our previous observations in malignant mesothelioma cell lines and primary specimens [47], points to an additional role for CSF-1R which complement its better characterized function at promoting development and survival of tumor-associated macrophages [70–75]. Unraveling a tumor-specific expression/function of the receptor has been facilitated by our use of mice bearing a deeply defective tumor microenvironment and of *in vitro* grown cell lines. However, despite such a limitation of our model, we did not observe, in the TKI-treated mice, correlation between the reduction of CD45^pos^ tumor infiltrating cells and the effect on tumor growth. Clearly, it remains to be addressed how the double effects of the TKI at inhibiting CSF-1R expressed by both tumor-infiltrating immune components and by the tumor cells is balanced in more clinically relevant settings and how this can be exploited, therapeutically.

The present work leaves some open questions. Our observations suggest a role for CSF-1R in modulating the chemotherapy-induced stress response. Of note, the CSF-1R^pos^ cells represent only a fraction of the entire tumor cell population. However, we observed a “collective’ effect on the resistance of the entire cell culture when reducing the levels and/or the activity of the receptor. This raises the possibility that paracrine mechanism(s) may underlie the ability of the CSF-1R^pos^ cells to propagate protumorigenic properties to adjacent, CSF-1R^neg^ cell subpopulations. We and others have shown that complex secretome rearrangements arise in chemo-treated tumor samples and this orchestrates the emergence of chemoresistant cell subpopulations, fueled by the onset of a Senescence Associated Secretory phenotype in the chemosensitive cell subpopulations [29, 30, 76, 77]. Given that CSF-1 is increased by cisplatin treatment, and that the CSF-1R^pos^ cells are chemoresistant, important to evaluate which signals modulate the expression of CSF-1 and CSF-1R within the context of a stress induced tumor rearrangement and whether the CSF-1R ligand may work as a SASP cytokine.

Similar to many TKI belonging to this class, the JNJ-40346527 compound may not exhibit absolute specificity toward CSF-1R, as being weakly specific against c-KIT and more specific against FLT3, *in vitro* [48]. However, the lack of effect on the CSF-1R^neg^ NCI-H460 cells *in vitro* and the correlation between the inhibition of tumor growth and the changes in the number of the tumor CSF-1R^pos^ cells *in vitro* and *in vivo*, suggest some degree of specific target engagement. Encouragingly enough, the mentioned TKI has been already evaluated in a recently developed phase 1/2 study directed toward relapsed or refractory classical Hodgkin lymphoma (cHL), which, as mentioned earlier, exhibits lineage appropriate expression of CSF-1R. In this study, some therapeutic efficacy was observed for the monotherapy treatment and very mild toxicity was shown even in the patients administered with TKI even at very high dosages [49]. Importantly, in the mentioned study no combination of JNJ-40346527 -with chemotherapy was attempted. Our study shows how the CSF-1R TKI inhibitor shows synergy with cisplatin *in vivo*. This correlates with the observation that the CSF-1/CSF-1R system is stimulated by stress –induced chemotherapy (as shown by the upregulation of both ligands and receptor in cisplatin treated samples), resulting in the persistence of CSF-1R expressing cells in cisplatin-treated samples, *in vitro* and *in vivo*. Thus, it is possible that, in a combination treatment, the TKI may exhibit superior efficacy as compared to single treatment. This may pave the way to further *in vivo* studies.

## MATERIALS AND METHODS

### Cell lines and culture conditions

The human lung cancer cell lines A549, NCI-H1299, H1975, CALU-1, NCI-H157, NCI-H358, NCI-H460 were from ATCC (Manassas, VA, USA). All the cell lines were Mycoplasma free and used within passages 2–8 from thawing. Cells were cultured as monolayers at 37°C and 5% CO_2_ in DMEM/F12+GLUTAMAX supplemented with 10% non-heat inactivated FBS (fetal bovine serum) (Life Technologies, Gran Island, NY USA).

### Reagents

Cisplatin (Selleckchem, Texas, USA) was dissolved according to the manufacturer's instructions. JNJ-40346527 was kindly provided by Janssen Research & Development and dissolved in DMSO. For *in vivo* studies, the JNJ-40346527 was dissolved in Methocel, 0.5%, before being used for oral gavage, according to the provider's instructions.

### ALDH activity assay

The ALDEFLUOR kit (Stem Cell Technologies, Vancouver, Canada) was used according to the manufacturer's instructions. ALDH-positive cells were defined as the cells that displayed greater fluorescence compared with a control staining reaction containing the ALDH inhibitor, DEAB (diethylaminobenzaldehyde), upon addition of the synthetic ALDH substrate BAAA. In some experiments, dead cells positive to SYTOX Red Dead Cell Stain (Life Technologies, Grand Island, NY) were excluded.

### Flow cytometry

Cells were detached by PBS1X/EDTA 2mM andfixed with 4% PFA (10 min on ice), washed twice with PBS1X and resuspended for antibody staining at 1 × 10^6^ cells/100 uL in PBS1X/BSA 1%. Live /dead cell discrimination was performed with the SYTOX-orange dye (Thermo Scientific), according to the manufacturer instructions. Gates (in the live cell population) were drawn in order to exclude > 99% of background staining (based on the isotype-stained samples). Data were acquired using a FACS CALIBUR instrument (BD Biosciences) and analysis was performed by using Summit 5.0.0 (Dako, Agilent Technologies, CA, USA). For CSF-1R staining, anti-CD115-APC conjugated and its highly adsorbed isotype matched control (Biolegend) were used.

### Cytokine quantification

ELISA-based cytokine quantification kits for CSF-1 (Abnova, Taipei City, Taiwan) and IL-34 (BioLegend, CA, USA) secreted in the conditioned media were commercially available.

### siRNAs

Silencer predesigned siRNA CSF-1 and siRNA IL-34 (Ambion-Life Technology, Foster City, CA, USA) were transfected into lung cancer cells using Lipofectamine 2000 (Invitrogen-GIBCO) according to the manufacturer's instructions.

### RNA extraction

Total RNA was extracted using the RNAeasy minikit (Qiagen, Hilden, Germany).

### cDNA synthesis and gene expression

The first-strand cDNA was synthesized according to manufacturer's instructions (High Capacity RNA-to-cDNA Kit; Applied Biosystems, Foster City, CA, USA). Gene expression was measured by real-time PCR using the SYBR Green dye (Applied Biosytems) on a StepOne Instrument (Applied Biosytems). qPCR primers are reported in Supplementary Table S1 and were previously described [47, 62, 78]. PPIA was used as an endogenous control.

### Colony forming assay (CFA)

Lung cancer cell lines were grown to 70% confluence and pulse- treated with the indicated drugs or transfected as indicated. 16 hrs later, cells were detached and seeded at 500–1500 cells/well into 6-well dishes in drug-free media (2 ml medium /well). Fresh medium (25%) was added every three days. Colonies were stained with crystal violet (SIGMA) and colonies (> 50 cells) counted after 7–14 days (this wide range reflects differences in the proliferation of the colonies for each lung cancer cell line). For 3D clonogenic assays, the cells were plated in anchorage independent and serum free conditions in DMEM-F12/1:1 + Glutamax supplemented with BSA and EGF(10ng/ml) and FGF-2(10 ng/ml) (Life Technologies) as previously described [56].

### Cell lysis, immunoprecipitation and Western blotting

Briefly, cells were lysed in cell lysis buffer: 50 mM Tris-HCl (pH 8), 2% SDS, 50 mM NaCl, 1 mM EDTA, and 10% glycerol, supplemented with protease and phosphatase inhibitors (Roche), to generate total cell extracts. For the western blotting the following antibody was used: rabbit anti-CSF-1R (C-20) (Santa Cruz Biotechnology); anti-human CSF-1R antibody (1:800, HPA012323) (SIGMA-Aldrich, Milan, Italy); anti-phospho-CSF-1R(tyr723) antibody (Cell Signaling); mouse anti-TUBULIN (Santa Cruz Biotechnology) was used as a loading control. For immunoprecipitation studies, a mouse monoclonal antibody against CSF-1R was used (D-8, Santa Cruz Biotechnology). For the chemiluminescent detection of the secondary antibodies, a Western Bright ECL HRP substrate (ADVANSTA, Menlo Park, CA, USA) was used.

### Animal studies

Suspensions of 5 × 10^6^ H1299 cells were injected subcutaneously in PBS1X/Geltrex (BD Bioscience) into 5-weeks-old male NOD/SCID mice (Charles River, Italy). Body weight and clinical signs of the mice were determined every 7 days. Intravital Imaging (IVIS) was performed at day 9, 23, 35, 42 after cell injection (pi). The values at day 9 (when tumors were already palpable) were used to assign each mice to homogeneous groups. Mice were treated intraperitoneally with vehicle (methocel, 0.5%), cisplatin (4 mg/kg, once a week/14 days, started at day 16 pi), JNJ-40346527 (20 mg/kg by oral gavage, QD, 16 days, started at day 14 pi), cisplatin + JNJ-40346527 (4 mg/kg + 20 mg/kg by IP injection and oral gavage, respectively). All animal work was performed in accordance with NYU guidelines and upon IACUC approval.

### Tumor disaggregation

Freshly excised tumors were manually minced before enzymatic disaggregated for 2 hrs with Accutase (Stem Cell technologies, Vancouver, CA) and pooled. Cell proliferation was assessed by quantification with Ki-67 immunohistochemistry on cytospin sections of the disaggregated tumors with a anti-human Ki-67 antibody (1:300, clone MIB-1) (Dako, CA, USA). Positive cells were scored by visual examination of 10 randomly chosen fields containing at least 100 cells. For FACS analysis of the tumor-derived cells, anti-human CSF-1R antibody (1:200, HPA012323) (SIGMA-Aldrich, Milan, Italy) and anti-mouse CD45 antibody (1:100, ABCAM, Cambridge, UK) were used

### Statistical analysis

One-way analysis of variance with Tukey's post hoc corrections-comparing the mean of each group with the mean of every other group or Student's *t-test* (comparing each sample to its control or, when indicated, to other samples within the same group). Statistical significance was defined as *p* < 0.05 except where indicated. The GraphPad software (GraphPad, San Diego, CA) was used for all the statistics.

## SUPPLEMENTARY MATERIALS FIGURES AND TABLE


