# Supplementary material for: Inhibition of the colony-stimulating-factor-1 receptor affects the resistance of lung cancer cells to cisplatin
Source: Oncotarget. 2016 Jul 28;7(35):56408–21. doi: 10.18632/oncotarget.10895 (PMC5302923; doi:10.18632/oncotarget.10895)
Supplement: Supplementary file 1 [file oncotarget-07-56408-s001.pdf]

Inhibition of the colony-stimulating-factor-1 receptor affects the resistance of lung cancer cells to cisplatin

Supplementary Materials

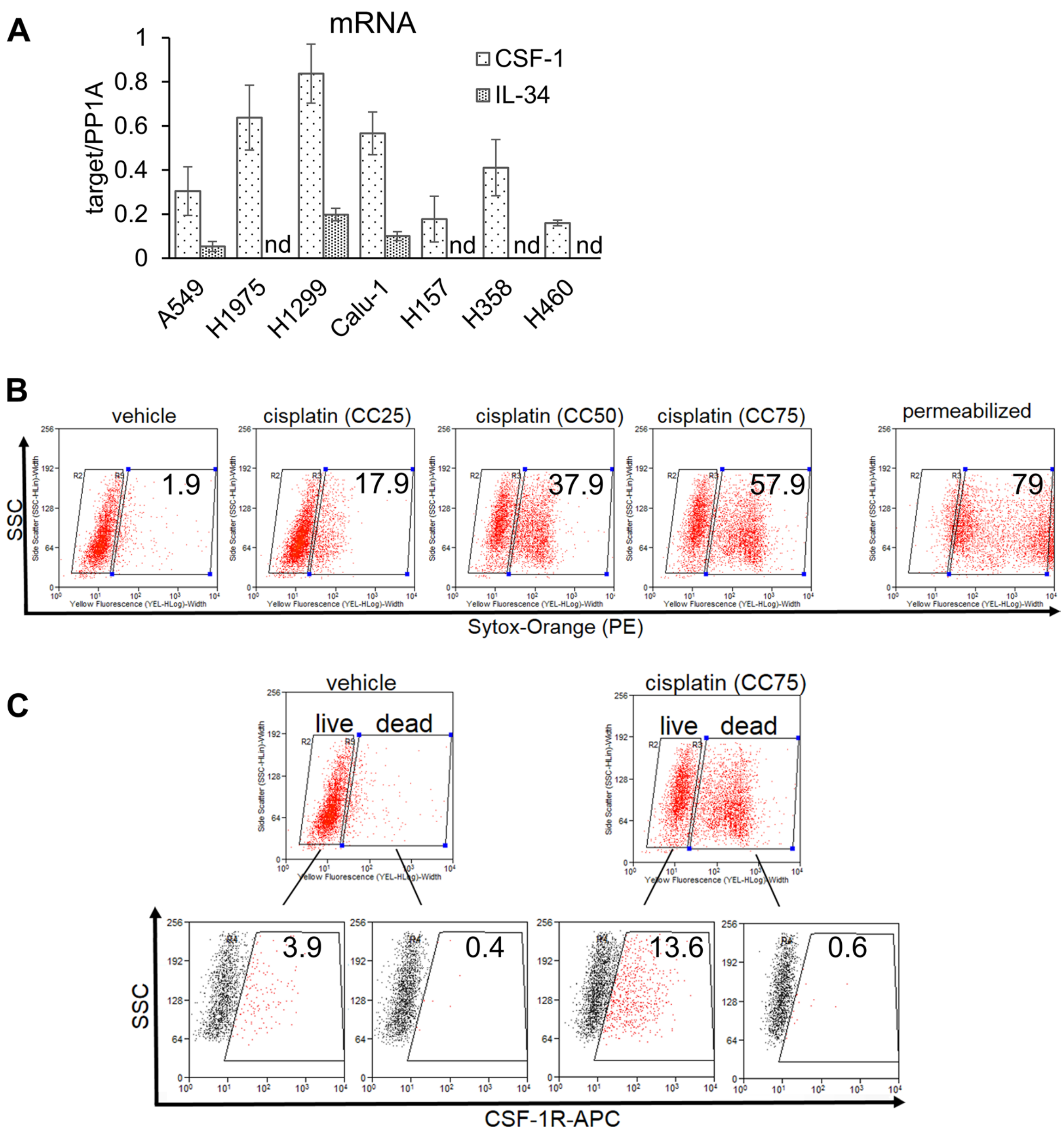

Supplementary Figure S1: (**A**) Lung cancer cell lines express CSF-1 and IL-34 mRNA. **(A)** Expression of CSF-1 and IL-34 mRNA (normalized to PP1A) as assessed by quantitative PCR. Mean ± SE of two experiments. **(B–C)** The CSF-1R^{pos} cells survive cisplatin treatment. **(B)** Representative dot plots of H1299 cells treated for 72 hrs with the indicated doses of cisplatin and stained with the cell impermeant dye Sytox-Orange before FACS analysis. As a positive control for the staining, H1299 treated with Triton X-100 for 10 min were stained (permeabilized). (C) Representative dot plots indicating the percentage of CSF-1R^{pos} cells in Sytox-negative (live) and Sytox-positive (dead) cells, respectively, after treatment with vehicle or cisplatin as from Supplementary Figure S1B.

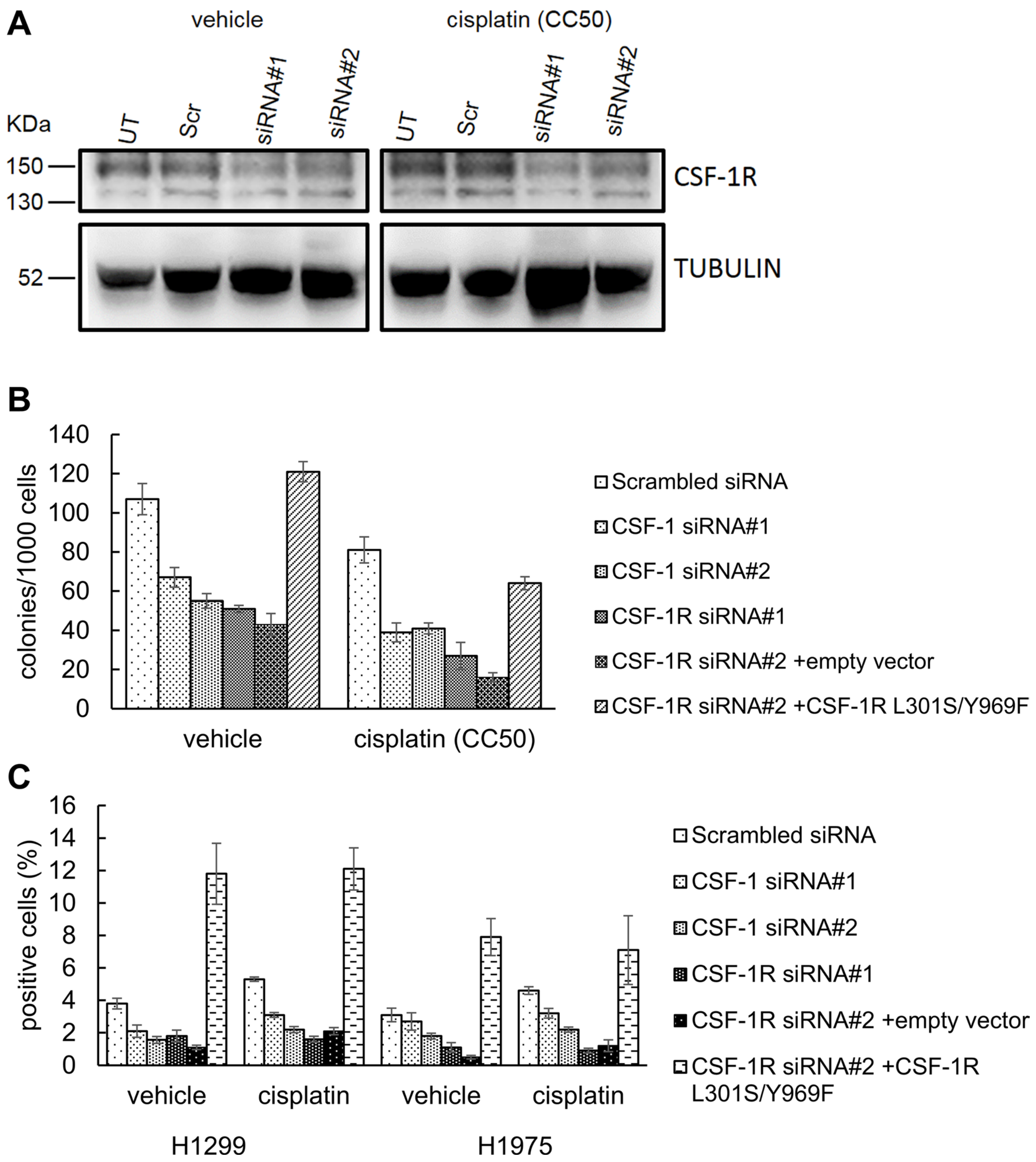

Supplementary Figure S2: (**A**) Knocking down of CSF-1R affects the clonogenicity and resistance to cisplatin of lung cancer cell lines. (A) Representative western blottings of whole cell lysates from H1299 cells untransfected (UT) or transfected with a scrambled-control and CSF-1R targeting- siRNAs, and treated with vehicle or cisplatin for 72 hrs. Staining for tubulin was used as a loading control. (**B**) Briefly, H1299 cells transfected with CSF-1R targeting siRNAs, were treated 48 hrs later with vehicle- or cisplatin (CC_{25})-for 16 hrs, and seeded at clonal density. Additionally, cells transfected with the CSF-1R targeting siRNA#2 were co-transfected with either an empty vector or a vector coding for a constitutively active CSF-1R (L301S/Y969F). Histograms reporting the number of the formed colonies 7–10 days after seeding**.** Mean ± SE of three independent experiments. (**C**) Histograms reporting the number of CSF-1R^{pos} cells in H1299 and H1975 cells treated as from Supplementary Figure S2B, as assessed by FACS. Mean ± SE of two independent experiments.

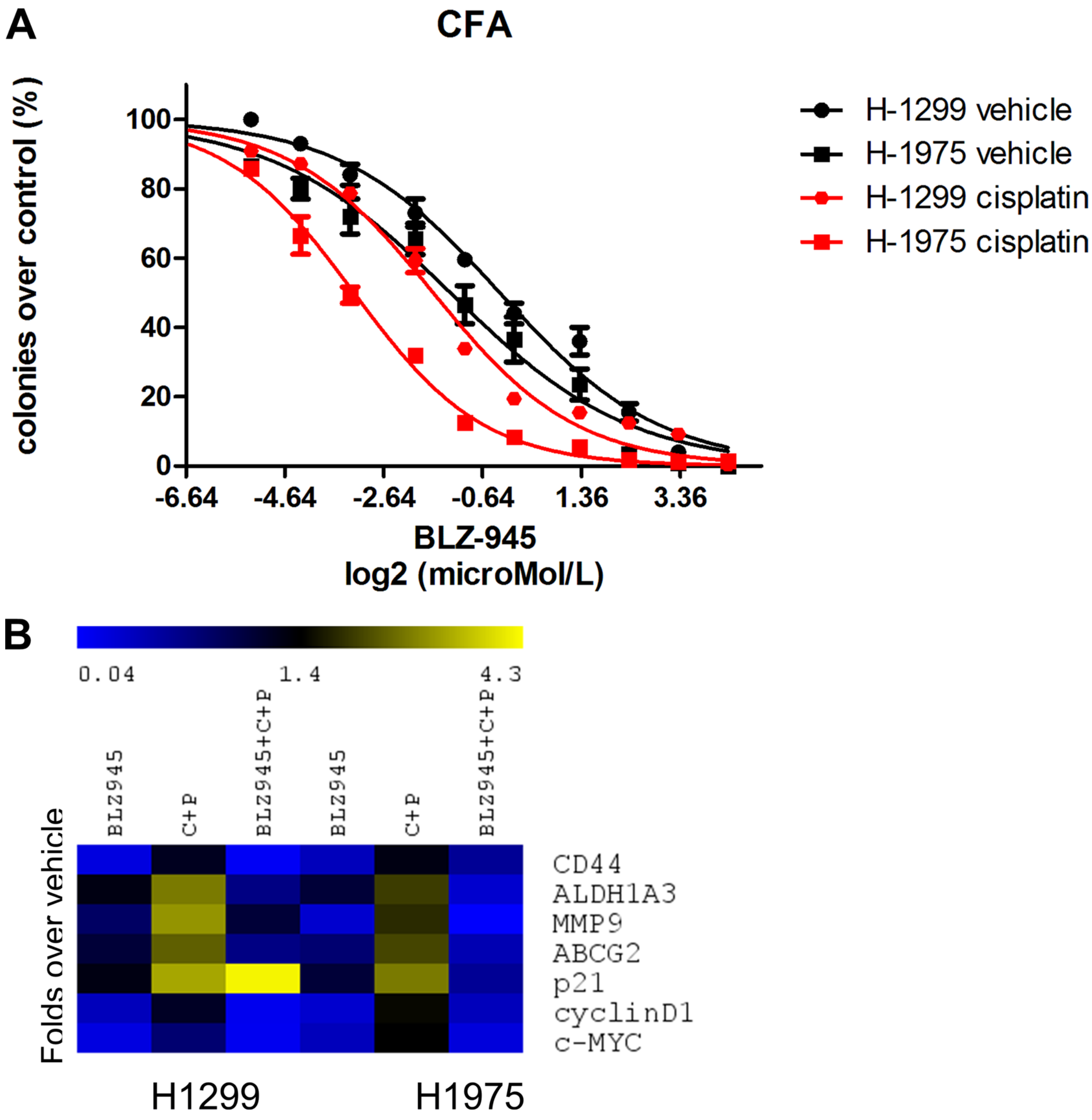

Supplementary Figure S3: (**A**) Treatment of lung cancer cell lines with BLZ-945 affects clonogenicity and expression of EMT genes. (A) Graph reporting the percentage of colonies (over vehicle-control) of H1299 and H1975 cells treated for 24 hrs with BLZ-945 at the indicated doses, in the presence of vehicle or cisplatin (CC_{25}), before seeding at clonal density. The formed colonies were stained 7-9dd later. Mean ± SE of two independent experiments. (**B**) Heatmap. Relative expression levels of proliferation- and EMT-associated genes in H1299 and H1975 cells treated with BLZ-945 (CC_{50}), C + P or both for 24 hrs, as assessed by quantitative PCR.

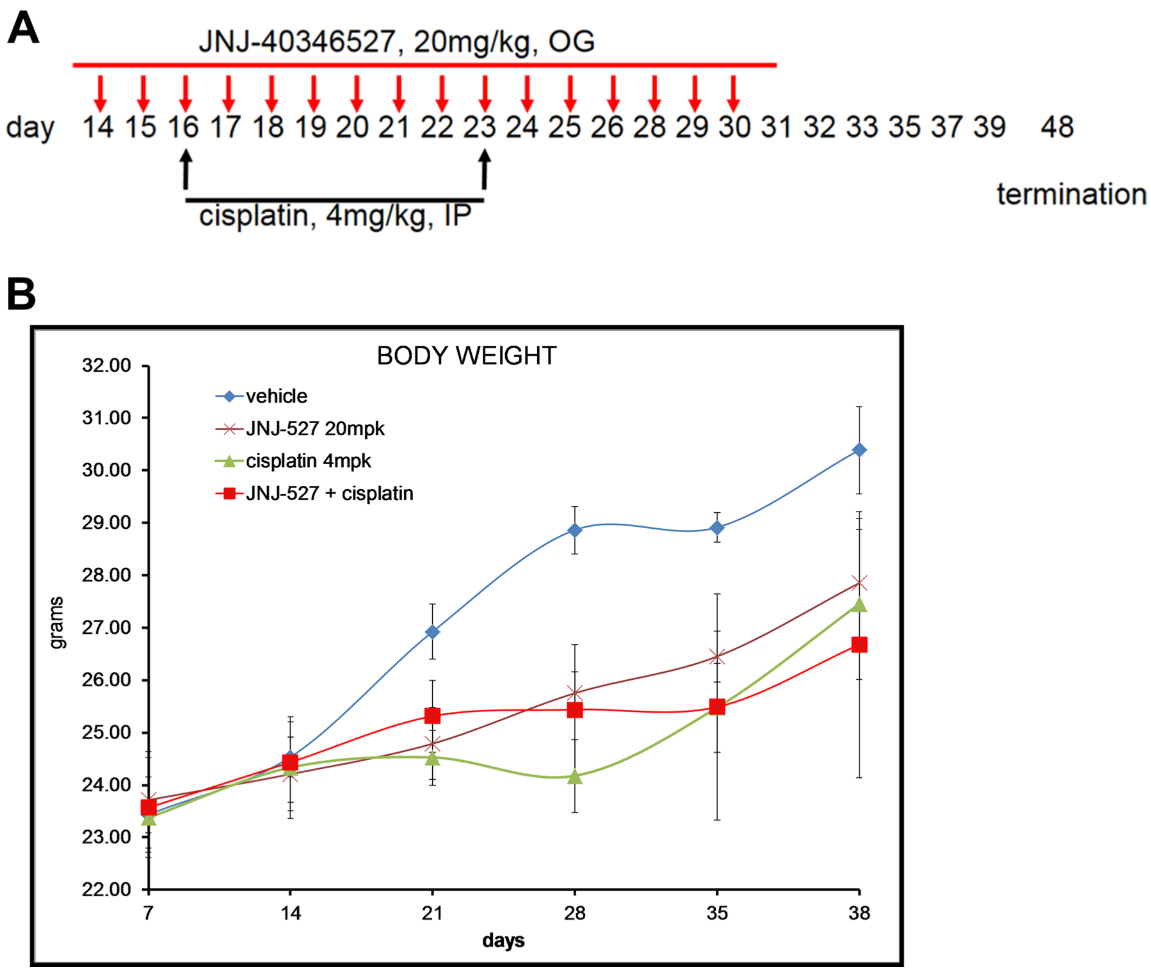

Supplementary Figure S4: (**A**) Scheme of the *in vivo* studies. Treatment started at day 14 (when the average tumor volume was ≥ 100 mm^3). Study was terminated at day 48, when tumors were excised, disaggregated and pooled before being processed for FACS. (**B**) A transient weight loss is induced by both cisplatin and JNJ-40346527 treatment. Graph reporting the average body weight of each group of mice as from Figure 4. The red bar indicates the period of treatment. Mean ± SE for each group of mice is reported.

Supplementary Table S1: List of the primers used in this study for the quantitative PCR assays

CSF-1R forward<br>CSF-1R reverse	GAATGACTCCAACTACATTGTC<br>GTGTAGACAGTCAAAGATG
CSF-1 forward<br>CSF-1 reverse	GCTGTTGTTGGTCTGTCTC<br>CATGCTCTTCATAATCCTTG
IL-34 forward IL<br>34 reverse	AAACAAAGCTCCGTCCTAAACTG<br>GCCGCATACTGCAATGAGG
OCT4 forward<br>OCT4 reverse	GGGAGATTGATAACTGGTGTGTT<br>GTGTATATCCCAGGGTGATCCTC
SOX2 forward<br>SOX2 reverse	TACAGCATGTCCTACTCGCAG<br>GAGGAAGAGGTAACCACAGGG
NANOG forward<br>NANOG reverse	TTTGTGGGCCTGAAGAAAACT<br>AGGGCTGTCCTGAATAAGCAG
C-MYC forward<br>C-MYC reverse	GTCAAGAGGCGAACACACAAC<br>TTGGACGGACAGGATGTATGC
VIMENTIN forward<br>VIMENTIN reverse	AGTCCACTGAGTACCGGAGAC<br>CATTTCACGCATCTGGCGTTC
ABCG2 forward<br>ABCG2 reverse	ACGAACGGATTAACAGGGTCA<br>CTCCAGACACACCACGGAT
ALDH1A3 forward<br>ALDH1A3 reverse	TCTCGACAAAGCCCTGAAGT<br>TATTCGGCCAAAGCGTATTC
MMP9 forward<br>MMP9 reverse	TGTACCGCTATGGTTACACTCG<br>GGCAGGGACAGTTGCTTCT
CD44 forward<br>CD44 reverse	AAGGTGGAGCAAACACAACC<br>AACTGCAATGCAAACTGCAAG
P21 forward<br>P21 reverse	TGTCCGTCAGAACCCATGC<br>AAAGTCGAAGTTCCATCGCTC
PPIA forward<br>PPIA reverse	TCTGAGCACTGGAGAGAAAGG<br>GGAAAACATGGAACCCAAAGG
Cyclin D1 forward<br>Cyclin D1 reverse	TTCGGGATGATTGGAATAGC<br>TGTGAGCTGGCTTCATTGAG
